# Whole-Genome Analysis of *Priestia aryabhattai* WJ45 Reveals a Genetic Repertoire Associated with Enhanced Wheat Germination and Early Seedling Growth Under Salt Stress

**DOI:** 10.3390/microorganisms14030605

**Published:** 2026-03-09

**Authors:** Ji-In Woo, Min Young Back, Ho-Jun Gam, Ju-Yeon Ha, Sang-Mo Kang, In-Jung Lee

**Affiliations:** Department of Applied Biosciences, Kyungpook National University, Daegu 41566, Republic of Korea; wjxsj99@knu.ac.kr (J.-I.W.); miny4310@knu.ac.kr (M.Y.B.); 2023001144@knu.ac.kr (H.-J.G.); hjy4726@knu.ac.kr (J.-Y.H.); sangmo@knu.ac.kr (S.-M.K.)

**Keywords:** whole genome sequencing, *Priestia aryabhattai*, plant growth-promoting bacteria, salt stress

## Abstract

Salinity stress constitutes a major environmental constraint impeding crop establishment by limiting water uptake and disrupting osmotic homeostasis during seed germination and early growth. Plant growth-promoting bacteria (PGPB) offer as a sustainable and cost-effective strategy to mitigate these limitations in agricultural systems. In this study, whole-genome analysis of the salt-tolerant PGPB *Priestia aryabhattai* WJ45 identified its genomic potential for PGP and salinity adaptation, alongside evaluations of wheat germination under saline conditions. Genome analysis revealed that strain WJ45 harbors a coordinated set of genes associated with key plant growth-promoting traits, including exopolysaccharide production, phosphate solubilization, and siderophore biosynthesis, as well as genes involved in Na^+^/K^+^ transport and osmolyte metabolism. Consistent with these genomic predictions, germination assays demonstrated that WJ45 treatment increased the germination rate by 13.1%, under salt stress compared with the non-inoculated control, while coleoptile, radicle lengths, and fresh weight were enhanced by 17.0%, 15.7%, and 53.2%, respectively, indicating improved early seedling establishment. Collectively, these findings demonstrate that WJ45 possesses a genome-encoded capacity to facilitate crop establishment under saline conditions. While further seedling and large-scale evaluations are warranted, this study underscores the potential of this genome-informed microbial resource to enhance early plant growth and resilience in salt-affected environments.

## 1. Introduction

The rapid growth of the global population has highlighted the urgent need to ensure global food security. It is estimated that global agricultural production will need to increase by more than 50% to reliably support a world population projected to exceed 9 billion by 2050 [1]. To address these demands, chemical fertilizers and pesticides have been widely adopted as relatively straightforward and effective tools for improving agricultural productivity. In line with these efforts to ensure food security, broader strategies are being explored, including the utilization of plant biodiversity for biostimulants and sustainable protein production [2,3]. Nevertheless, the extensive application of chemical inputs has been consistently criticized for their adverse environmental impacts [4]. Prolonged use of these chemical inputs can modify the physical and chemical characteristics of soils, leading to soil health deterioration, and has been reported to progressively intensify soil salinization in intensive agricultural and irrigation-based cropping systems [5,6]. Soil salinity decreases soil water potential, thereby restricting plant water uptake, and causes ionic imbalances and disruption of cellular homeostasis as a result of excessive ion accumulation, ultimately exerting toxic effects on plants [7]. This salt stress impairs plant physiological functions from germination through subsequent growth and development, constituting a significant environmental limitation on crop productivity [8]. Among the major crops affected, wheat (*Triticum aestivum* L.) is of particular concern as a staple food for a substantial portion of the global population [9]. Wheat is generally regarded as a salt-sensitive crop, with its vulnerability being particularly pronounced during early developmental stages such as germination and seedling establishment [10,11,12]. Conventional approaches to address this problem have often relied on increasing chemical amendments to temporarily sustain crop yields; however, this fails to address the underlying salinity issues and, instead, intensifies salt accumulation in the soil, thereby exacerbating degradation and creating a self-perpetuating negative cycle [13]. Consequently, there is a pressing need for fundamental and sustainable strategies, such as biological interventions, that can restore soil health while providing long-term stress tolerance.

Given the inherent limitations of conventional chemical amendments in mitigating the complex physiological effects of salinity, the use of plant growth-promoting bacteria (PGPB) has emerged as a pivotal biological strategy for sustainable soil and crop management. Through symbiotic interactions, PGPB can substantially enhance plant resilience and growth under stress conditions [14,15,16]. PGPB promote plant growth via multiple mechanisms, including nitrogen fixation, mineral solubilization, and phytohormone production, making them potential substitutes for or complements to conventional chemical inputs [17]. In particular, under salt stress conditions, PGPB have been reported to enhance plant stress tolerance by producing osmoprotective compounds, forming physical protective barriers on the seed surface, and stimulating antioxidant defenses [18]. However, under field-relevant conditions, the stable expression of these beneficial functions requires the selection of stress-resilient strains and a clear understanding of their underlying mechanisms of action.

Whole-genome sequencing (WGS) provides a robust framework for predicting the functional potential and environmental adaptability of PGPB at the genetic level [19]. While experimental validation remains indispensable, these genomic methodologies enable a more comprehensive understanding of the molecular mechanisms governing plant–microbe interactions. Consequently, the present study aimed to isolate and select a PGPB strain demonstrating tolerance to saline conditions and to investigate the genetic determinants underlying its salt stress tolerance and plant growth-promoting characteristics through whole-genome analysis. This study provides a scientific basis for microbial-based strategies designed to alleviate salt stress and promote sustainable agricultural practices.

## 2. Materials and Methods

### 2.1. Isolation of Salt Tolerance Plant Growth-Promoting Bacteria

Bacterial strains were isolated from rhizosphere soil samples collected from a weed community adjacent to an agricultural field in Daegu, Republic of Korea (35.9533° N, 128.6909° E), characterized by a history of long-term successive cropping. This location was chosen due to the presence of osmotic stress caused by the accumulation of fertilizer-derived salts, making it a suitable source for isolating salt-tolerant PGPB. One gram of rhizosphere soil was suspended in 0.85% (*w*/*v*) NaCl solution, serially diluted, and plated onto tryptic soy agar (TSA) plates, followed by incubation. Morphologically distinct colonies were selected to obtain bacterial isolates. The selected isolates were cultured in tryptic soy broth (TSB) supplemented with various NaCl concentrations (0, 3, 5, 7, and 10%, *w*/*v*). and their salt tolerance was assessed by measuring optical density at 600 nm (OD600) at 4 days after incubation.

### 2.2. Evaluation of Plant Growth-Promoting Traits

The selected salt stress–tolerant isolates were evaluated for plant growth-promoting (PGP) traits using established methodologies:

**Exopolysaccharide (EPS) production** was assessed on Congo red agar medium following the protocol described by [20]. The medium comprised Luria–Bertani broth (25 g L^−1^), sucrose (5% (*w*/*v*)), Congo red (0.8 g L^−1^), and agar (2% (*w*/*v*)). The formation of black colonies on this medium was considered indicative of EPS secretion.
**Siderophore production** was determined using Chrome Azurol S (CAS) agar according to the method outlined by [21]. The CAS agar medium was prepared by combining nutrient broth (8 g L^−1^), PIPES buffer (27.216 g L^−1^), and agar (2% *w*/*v*), adjusted to pH 6.5, followed by the addition of the CAS reagent. The CAS reagent was prepared by dissolving CAS and hexadecyltrimethylammonium bromide in distilled water, followed by the addition of an Fe^3+^ solution prepared in dilute HCl and sterilization by autoclaving. The appearance of an orange halo surrounding bacterial colonies was interpreted as positive siderophore production.
**Phosphate solubilization activity** was evaluated on Pikovskaya’s agar medium as described by [22], which contained glucose (10 g L^−1^), yeast extract (0.5 g L^−1^), (NH_4_)_2_SO_4_ (0.5 g L^−1^), NaCl (0.3 g L^−1^), KCl (0.3 g L^−1^), FeSO_4_ (0.03 g L^−1^), MgSO_4_ (0.3 g L^−1^), MnSO_4_ (0.03 g L^−1^), Ca_3_(PO_4_)_2_ (5 g L^−1^), and agar (2% (*w*/*v*)). The formation of a clear halo surrounding colonies indicated phosphate solubilization.
**Indole-3-acetic acid (IAA) production** was assessed using culture supernatants obtained after incubation in TSB for 72 h. The supernatant was mixed with Salkowski’s reagent and incubated for 30 min. The development of a pinkish-red coloration was considered indicative of IAA production [22].

For a comparative evaluation of these PGP traits, specific scoring criteria were applied. For EPS, siderophore, and phosphate solubilization, isolates were categorized as “positive” if a halo or distinct colony margin was visible, and “superior” if the halo radius exceeded 2 mm beyond the colony boundary. In the case of IAA, quantitative thresholds were set at OD530 0.1 ≥ for “positive” and OD530 ≥ 0.25 for “superior” classifications. Based on this stringent screening process, strain WJ45 was selected for subsequent experiments.

### 2.3. Genomic Analysis and Functional Characterization

Genomic DNA was extracted from WJ45 cells cultured in TSB at 28 °C with shaking at 150 rpm for 24 h. Genomic DNA isolation was performed using the Wizard Genomic DNA Purification Kit (Promega, Madison, WI, USA) according to the manufacturer’s protocol. DNA purity was evaluated using a NanoDrop 2000 UV–Vis spectrophotometer (Thermo Fisher Scientific, Waltham, MA, USA), and DNA concentration was measured with a Qubit 2.0 fluorometer (Invitrogen, Waltham, MA, USA). The extracted DNA samples were stored at –80 °C until further analysis. Sequencing libraries were prepared using the Ligation Sequencing Kit V14 (SQK-LSK114; Oxford Nanopore Technologies, London, UK) in accordance with the manufacturer’s protocol, without a DNA size selection step. Whole-genome sequencing (WGS) was conducted on a MinION platform (Oxford Nanopore Technologies, London, UK) equipped with an R10.4.1 flow cell. FASTQ files were generated using Guppy version 4.4.1 (https://community.nanoporetech.com; accessed on 14 December 2025). Sequence reads were assembled de novo using Flye version 2.9.5, and assembly quality was assessed with Quality Assessment Tool for Genome Assemblies (QUAST) version 5.2.0. Genome annotation was performed using Rapid Annotations using Subsystems Technology (RAST), Prokka, and the NCBI Prokaryotic Genome Annotation Pipeline (PGAP) [23]. WGS and primary data processing of strain WJ45 were performed at the Next-Generation Sequencing Core Facility of Kyungpook National University, Republic of Korea. Functional annotation of the WJ45 genome was further carried out using the Clusters of Orthologous Groups (COG), Kyoto Encyclopedia of Genes and Genomes (KEGG), and Gene Ontology (GO) databases, with particular emphasis on genes associated with plant growth promotion, salt stress tolerance, and antioxidant activity [24]. In addition, secondary metabolite biosynthetic gene clusters related to the production of various classes of antimicrobial compounds were identified using antiSMASH (https://antismash.secondarymetabolites.org/; accessed on 17 December 2025).

#### 2.3.1. Molecular Identification of WJ45 Strain

Taxonomic identification of strain WJ45 was initially performed via 16S rRNA gene sequence analysis, which identified the isolate as *Priestia aryabhattai* (Accession number: PZ092777; Figure S1). To achieve higher taxonomic resolution, Average Nucleotide Identity (ANI) was calculated. Fourteen representative genomes of *P. aryabhattai* and its closely related species were retrieved from the NCBI database, with priority given to type strains and complete genome status to ensure high-quality comparisons. Pairwise ANI values were determined using FastANI (version 1.1). In accordance with established taxonomic standards, strains exhibiting ANI values above the 95–96% threshold were considered to belong to the same species.

### 2.4. In Vitro Seed Bioassay

#### 2.4.1. Preparation of Bacterial Inoculant and Biopriming

Wheat seeds (*Triticum aestivum* L. cv. Keumgang), a widely cultivated and well-characterized Korean cultivar, were obtained from the Upland Crop Development Division of the National Institute of Crop and Food Science (Miryang, Republic of Korea). For the in vitro seed bioassay, four treatment groups were established: (i) non-inoculated seeds with distilled water (DW), (ii) non-inoculated seeds with NaCl, (iii) seeds inoculated with strain WJ45 and treated with DW, and (iv) seeds inoculated with strain WJ45 and treated with NaCl. The bacterial inoculum used for the germination assay was prepared by culturing strain WJ45 in TSB at 28 °C for 24 h. Cells were harvested by centrifugation at 4000 rpm for 10 min and resuspended in sterile distilled water to a final concentration of approximately 10^8^ colony-forming units (CFU) per milliliter. Wheat seeds were surface sterilized with 3% sodium hypochlorite for 30 s and thoroughly rinsed with sterile distilled water. The sterilized seeds were soaked in sterile distilled water for 22 h and subsequently immersed in the WJ45 suspension for 2 h for seed priming, while control seeds were maintained in sterile distilled water. During this priming period, the stability of cell viability in the distilled water was confirmed by monitoring CFU changes, which showed no significant reduction (Table S1; *p* = 0.147). Sterile filter paper (Advantec No. 2, Toyo Roshi Kaisha Ltd., Tokyo, Japan) was placed in Petri dishes (90 × 15 mm) and moistened with 2 mL of either distilled water or 100 mM NaCl solution. 12 treated seeds were placed in each Petri dish, with six biological replicates per treatment. Seeds were incubated for 7 days in a plant growth chamber (JSPC-420C, JSR Corporation, Gongju, Republic of Korea) under controlled conditions of 20 °C, 60% relative humidity, 6850 lux light intensity, and a 16/8 h light/dark photoperiod. The number of germinated seeds was recorded daily at the same time for 7 days. Early growth parameters were measured 7 days post-incubation. The lengths of the radicle and coleoptile were determined by measuring from the seed attachment point to their respective tips. To ensure an accurate representation of seedling biomass, the seeds were removed prior to measuring the fresh weight.

#### 2.4.2. Germination Index

Germination-related parameters, including final germination percentage (GP), mean germination time (MGT), germination index (GI), coefficient of velocity of germination (CVG), mean daily germination percent (MDG), synchronization index (Z), and germination value (GV) were calculated using the following formulas [25]:

(1) $$GP=(\sum n_{i}/N)\times 100,$$

(2) $$MGT=\sum (n_{i}\times t_{i})/\sum n_{i},$$

(3) $$GI=\sum (n_{i}/t_{i}),$$

(4) $$CVG=\frac{\sum_{i=1}^{k}n_{i}t_{i}}{\sum_{i=1}^{k}n_{i}}\times 100,$$

(5) $$MDG=\frac{GP}{T_{n}},$$

(6) $$Z=\frac{\sum n_{i}\left(n_{i}-1\right)}{N\left(N-1\right)},$$

(7) GV=MDG×PV,

where n_i_ is the number of seeds germinated at time t_i_, t_i_ is the germination time (day), N is the total number of seeds sown, and PV is peak value or largest quotient obtained when all the cumulative germination percentages were divided by the respective time interval.

### 2.5. Statistical Analysis

All experiments were conducted using a completely randomized design. Germination-related parameters were analyzed using at least three independent biological replicates per treatment (n = 3), and seedling growth parameters were analyzed at the individual seedling level (n ≈ 30 per treatment). Data are presented as mean ± standard deviation (SD). Statistical differences among treatments were evaluated using one-way analysis of variance (ANOVA) followed by Duncan’s multiple range test (DMRT) using GraphPad Prism (version 10.6.1; GraphPad Software, San Diego, CA, USA). Outliers were identified and excluded using the interquartile range (IQR) method prior to statistical analysis. Statistical significance was determined at *p* < 0.05. Genome data processing and visualization were performed using R (version 2026.01.0, build 392) with the dplyr (version 1.2.0), tidyr (version 1.3.2), ggplot2 (version 4.0.2), circlize (version 0.4.17), and ComplexHeatmap (version 2.26.1) packages.

## 3. Results

### 3.1. Screening and Characterization of Salt Stress Tolerance PGPB

**Figure 1:**
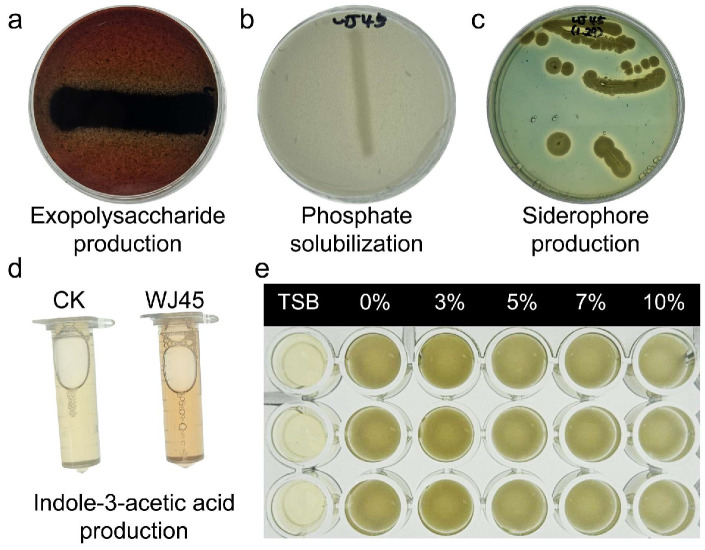
Phenotypic characterization of plant growth-promoting traits and salt stress tolerance in strain WJ45. (**a**) Exopolysaccharide production, (**b**) phosphate solubilization, (**c**) siderophore production, and (**d**) indole-3-acetic acid production were assessed using standard qualitative assays. (**e**) Salt tolerance was evaluated based on bacterial growth under increasing NaCl concentrations (0–10%), expressed as relative optical density compared with the non-salt control.

A total of 71 bacterial isolates were obtained from the collected soil samples and screened for salt stress tolerance. Among these, 28 isolates demonstrated the ability to grow on medium supplemented with 10% NaCl and were initially selected. Of the salt-tolerant isolates, strain WJ45 exhibited comparatively higher levels of EPS production, phosphate solubilization, and siderophore production than the other isolates, and was therefore selected for further analyses (Table 1 and Table S2; Figure 1).

### 3.2. Genomic Features and Taxonomic Identification of Strain WJ45

**Figure 2:**
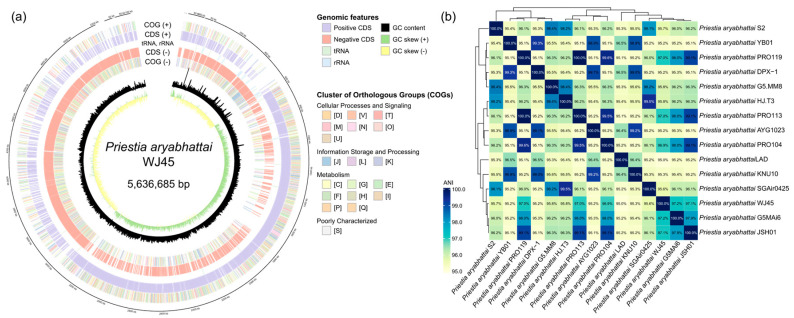
(**a**) Circular genome map of *Priestia aryabhattai* WJ45 showing coding sequences (CDSs), RNA genes, GC content, GC skew, and functional classification based on COG categories. (**b**) Average nucleotide identity (ANI) heatmap illustrating the genomic relatedness and intraspecific diversity of WJ45 compared with closely related *P. aryabhattai* strains.

To elucidate the genomic potential of strain WJ45 in relation to plant growth promotion and salt stress tolerance, WGS was conducted. The draft genome of strain WJ45 was assembled into nine contigs with a total length of 5,636,685 bp. The assembly exhibited high continuity, characterized by an N50 and maximum contig length of 5,264,238 bp. The individual contig sizes are 5,264,238, 117,210, 105,089, 57,054, 56,422, 12,960, 11,270, 8438, and 4004 bp. The genome harbored 5269 CDSs, 126 tRNA, and 40 rRNA genes, with an overall GC content of 37.87% (Figure 2a). The genome sequence of WJ45 has been deposited in the NCBI database (Accession number: JBUBQE000000000).

ANI analysis revealed that strain WJ45 shared more than 95% sequence identity with reference genomes of *Priestia aryabhattai*, supporting its classification within this species. Notably, WJ45 exhibited ANI values exceeding 97% with strains G5Mai6, JSH01, and PRO113, whereas a comparatively lower ANI value of approximately 95% was observed with strain LAD, representing the lowest genomic similarity among the analyzed genomes (Figure 2b).

### 3.3. Functional Annotation and Pathway Distribution of the WJ45 Genome

**Figure 3:**
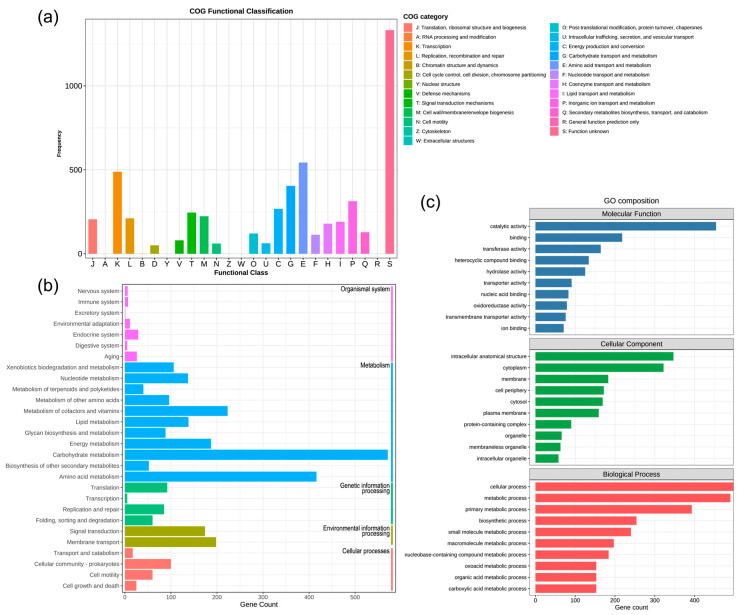
Functional annotation and classification of the *Priestia aryabhattai* WJ45 genome. (**a**) Clusters of Orthologous Groups (COG) classification of predicted coding sequences in the WJ45 genome. (**b**) Kyoto Encyclopedia of Genes and Genomes (KEGG) pathway classification of WJ45 genes across major functional categories. (**c**) Gene Ontology (GO) classification of WJ45 genes into molecular function, cellular component, and biological process categories.

Functional annotation of the WJ45 genome was performed using the COG, KEGG, and GO databases. Of the 5269 predicted genes in the WJ45 genome, 4775 were assigned to 19 functional categories in the COG database. Among these, 1332 genes (28%) were classified as function unknown (S). Within the functionally categorized genes, those involved in amino acid transport and metabolism (E) and transcription (K) were the most prevalent, comprising 543 genes (11%) and 488 genes (10%), respectively. These were followed by carbohydrate transport and metabolism (G; 404 genes, 8%) and inorganic ion transport and metabolism (P; 313 genes, 7%) (Figure 3a).

KEGG pathway analysis assigned 4775 genes to functional pathways. Among these, 2054 genes were associated with metabolism-related pathways, with the highest representation observed in carbohydrate metabolism (571 genes) and amino acid metabolism (416 genes). In addition, 372 genes were involved in environmental information processing pathways, including signal transduction (198 genes) and membrane transport (174 genes) (Figure 3b).

GO analysis classified 943 genes into three main categories: molecular function, cellular component, and biological process. Within the molecular function category, catalytic activity (454 genes) and binding (218 genes) were the most abundant terms. The major cellular component terms included intracellular anatomical structure (347 genes) and cytoplasm (322 genes). In the biological process category, cellular process (498 genes) and metabolic process (490 genes) were the most highly represented (Figure 3c).

Functional annotation of the WJ45 genome identified genes associated with key PGP traits (Table 2 and Table S3). Three genes involved in EPS production (*algA*, *pgaD*, and *bcsA*), five genes related to phosphate solubilization (*phoD*, *phoR*, *phoU*, *phoA*, and *phoB*), and nine genes associated with siderophore biosynthesis and transport (*asbA*, members of the *IucA*/*IucC* family, *entB*, two copies of *fhuC*, *fhuD*, and two copies of *fhuB*) were detected.

To examine the genetic basis of salt stress tolerance, genes involved in sodium (Na^+^) transport, potassium (K^+^) transport, osmolyte biosynthesis, and osmolyte transport were analyzed. The genome contained nine Na^+^ transport–related genes, including two copies of *nhaC* and the *mrpA–G* operon. Six genes associated with K^+^ ion homeostasis were identified, comprising three copies of *trkA*, two copies of *trkH*, and *ktrD*. In addition, six genes involved in osmolyte biosynthesis (*proA*, *proB*, and *proC*, each present in two copies) were detected. Multiple genes encoding compatible solute transport systems were also identified, including *proP*, two copies of *proV*, *proW*/*proX*, three copies of *opuAB*, and *opuD*. Furthermore, nine genes implicated in reactive oxygen species (ROS) scavenging and redox homeostasis were identified (Table 2 and Table S3).

AntiSMASH analysis identified a total of ten biosynthetic gene clusters (BGCs) in the WJ45 genome (Table S4). These included clusters associated with terpene and terpene-precursor biosynthesis, non-ribosomal iron-chelating siderophores (NI-siderophore), opine-like metallophores, RiPP-like compounds, and polyketide synthase families, including type III polyketide synthase (T3PKS) and highly reducing type II polyketide synthase (HR-T2PKS) clusters.

### 3.4. Effects of WJ45 on Wheat Germination Under Saline Conditions

**Figure 4:**
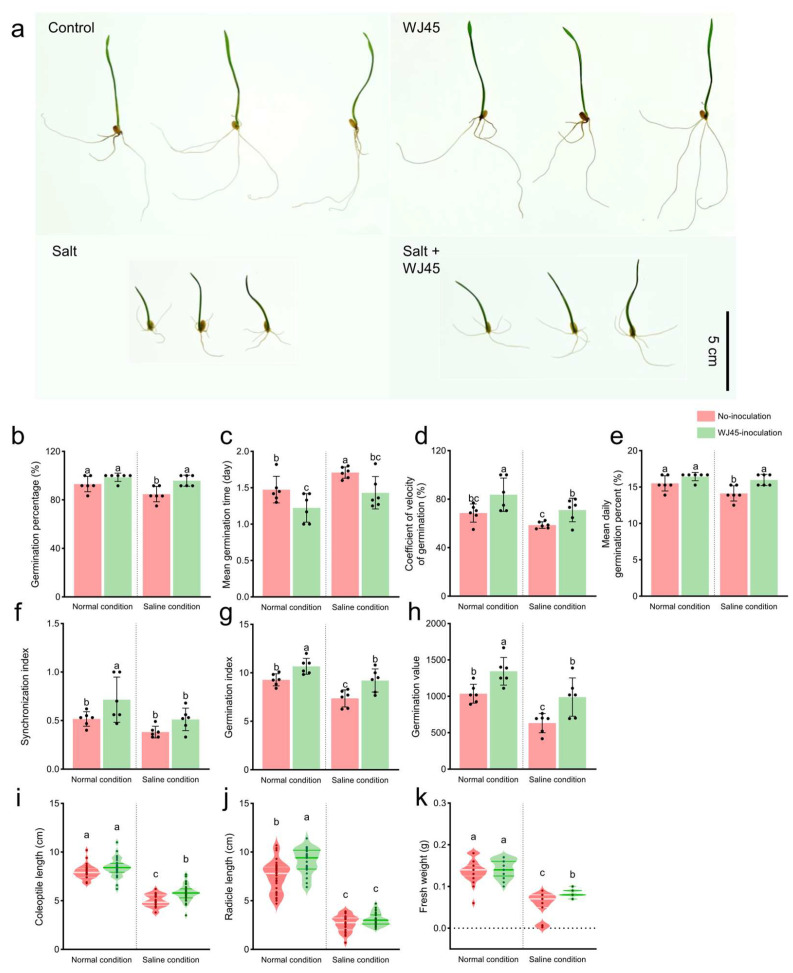
Effects of WJ45 priming on wheat germination and early seedling growth under salt stress. (**a**) Representative images of wheat seedlings. (**b**–**h**) Calculated germination-related indices: (**b**) germination percentage, (**c**) mean germination time, (**d**) coefficient of velocity of germination, (**e**) mean daily germination percentage, (**f**) synchronization index, (**g**) germination index, and (**h**) germination value. (**i**–**k**) Early growth parameters: (**i**) coleoptile length, (**j**) radicle length, and (**k**) fresh weight of wheat seedlings. Bars represent means ± SD (n = 6), and violin plots show the distribution of individual seedlings (n ≈ 30 per treatment). Different letters indicate statistically significant differences (*p* < 0.05).

Germination assays showed that WJ45 treatment significantly increased wheat germination and early seedling growth under saline conditions. Salt stress markedly inhibited germination and seedling establishment; however, biopriming with WJ45 effectively alleviated these salinity-induced inhibitory effects. Specifically, in wheat seeds subjected to salt stress, treatment with WJ45 resulted in increases in GP, MGD, CVG, Z, GI, and GV by 13.1%, 13.1%, 21.0%, 34.1%, 25.1%, and 56.5%, respectively. Conversely, MGT decreased by 16.4%, indicating that WJ45 not only improved overall germination performance but also enhanced the rate and synchronization of germination under saline conditions. Consistent with these results, the lengths of the coleoptile, radicle length and fresh weight were increased by 17.0%, 15.7%, and 53.2%, respectively, following WJ45 treatment under saline conditions (Figure 4; Table S5).

## 4. Discussion

Maintaining stable crop growth under variable soil conditions, particularly those associated with salt accumulation, remains a significant challenge for modern agriculture in the pursuit of sustainable food production. PGPB are increasingly recognized as a promising strategy to alleviate this constraint. In this study, genomic analysis was performed to identify genetic determinants associated with plant growth promotion and salt tolerance in the PGPB strain WJ45. In vitro assays using wheat seeds demonstrated that biopriming with WJ45 enhanced germination performance and improved stress tolerance during the early seedling establishment stage under saline conditions.

Based on ANI analysis derived from WGS, strain WJ45 was identified as *Priestia aryabhattai*, a species previously reported to exhibit plant growth-promoting properties and the ability to mitigate abiotic stress [26,27,28]. The functional analysis of the WJ45 genome presented in this study offers a genomic framework for interpreting the physiological characteristics described in prior research and further enhances our understanding of the plant–microbe interaction potential of *P. aryabhattai*.

The WJ45 genome contains multiple plant growth-promoting genes whose predicted functions align with the bioassay results obtained in this study. Traits such as EPS production, phosphate solubilization, and siderophore biosynthesis are widely recognized not only for their roles in biofertilizer efficacy but also for their ecological contributions to plant stress adaptation and tolerance under adverse environmental conditions [29,30].

EPS have been reported to stabilize the microenvironment surrounding seeds and emerging seedlings during microbial biopriming processes [31,32]. Previous studies have demonstrated that EPS-producing *Bacillus* species enhance wheat germination and early growth under salt stress [33]. While high EPS production is a well-established characteristic of *Priestia* and related *Bacillus* species [34,35,36], the precise structural composition underlying these EPS layers at the genomic level remain insufficiently characterized in many *P. aryabhattai* isolates. However, the chemical traits of EPS are critical as their functional roles depend on their structural composition. For instance, alginate-type EPS, characterized by high viscosity and water retention capabilities, may mitigate osmotic stress in the immediate vicinity of germinating seeds under saline conditions [37]. In the present study, the identification of diverse EPS-related genes in the WJ45 genome—including *algA*, *pgaD*, and *bcsA*—indicates that this strain possesses the genetic capacity to synthesize structurally diverse classes of EPS. This suggests a multifaceted protective mechanism mediated by its complex EPS repertoire, which is consistent with the significantly higher germination rates observed in seeds bioprimed with WJ45 under saline conditions. In addition to EPS production, the identification of genes associated with microbial colonization—including those involved in chemotaxis, motility, and cellular adhesion (Table S6)—demonstrates the genetic potential of strain WJ45 to establish both epiphytic and endophytic associations with wheat tissues. These results align with previous findings in various *Priestia aryabhattai* strains, which are known to form robust symbiotic relationships that enhance host stress resilience [38].

By comparison, inorganic ion supplementation facilitated by phosphate solubilization and siderophore production may have a limited impact during the initial stages of seed germination, as seeds predominantly depend on internally stored nutrients prior to root emergence [39,40]. However, during the subsequent seedling establishment phase, these traits may contribute to stabilizing the transition to early growth by enhancing nutrient acquisition following germination. Consistent with this notion, seeds bioprimed with WJ45 exhibited pronounced promotion of coleoptile elongation after germination. In addition, BGC analysis revealed that the WJ45 genome harbors not only NI-siderophore–related clusters but also opine-like metallophore clusters associated with metal chelation. Such metal uptake mechanisms may be linked to enhanced antioxidant enzyme activity, thereby facilitating the scavenging of ROS and alleviating oxidative stress induced under saline conditions [41].

Under saline conditions, the sustained survival and metabolic activity of PGPB are key prerequisites for the successful application of microbial biopriming, with ion homeostasis mechanisms playing a pivotal role in this process. The genome of strain WJ45 contains multiple genes implicated in Na^+^ extrusion and ionic balance, which is consistent with the stable survival phenotype observed under high-salinity conditions in this study. The *mrp* operon (*mrpA–G*) encodes a multisubunit Na^+^/H^+^ antiporter complex, while *nhaC* encodes a single-component Na^+^/H^+^ antiporter involved in regulating intracellular Na^+^ concentrations during salt stress [42,43]. Furthermore, the t*rk*/*ktr* system constitutes a widely conserved K^+^ transporter in bacteria, facilitating K^+^ uptake from the external environment and thereby contributing to the maintenance of intracellular Na^+^/K^+^ homeostasis [44]. Collectively, these Na^+^ extrusion- and K^+^ uptake-based ion homeostasis systems likely provide a mechanistic basis for the ability of WJ45 to survive and maintain physiological activity under saline conditions.

Biological systems employ common strategies to mitigate osmotic stress, among which osmoprotectants represent an important component. These low-molecular-weight organic compounds accumulate in response to osmotic stress, maintaining intracellular osmotic balance and safeguarding cellular functions [45]. In this context, the WJ45 genome is predicted to produce terpenoid compounds, which can indirectly support osmoprotection by interacting with the metabolic pathways that regulate osmolyte accumulation. Furthermore, these terpenes help maintain membrane integrity and fluidity under the osmotic and ionic stress conditions caused by salinity, thereby preserving cellular function [46]. Consistent with these mechanisms, the WJ45 genome also possesses genes associated with proline biosynthesis and proline/glycine betaine transport systems. This coordinated accumulation may facilitate osmotic adjustment, enhance antioxidant defenses, and regulate cellular turgor [47]. Collectively, these processes may have contributed positively to wheat germination under salt stress.

Despite the physiological mechanisms involved, the growth enhancement induced by WJ45 biopriming was less pronounced in the radicle than in the coleoptile under salt stress conditions. This difference likely reflects the inherent salt-tolerance strategies of wheat plants. Under salinity stress, plants typically modify their internal signaling pathways to reduce the root-to-shoot ratio, a strategic adaptation aimed at minimizing root surface exposure to toxic ions [48]. Although these mechanisms indicate that WJ45 promotes early growth, the seedlings remain subjected to high salinity, thereby sustaining an internal signaling network that favors shoot development. Consequently, the relatively modest improvement observed in radicle growth suggests that the plants continue to prioritize their intrinsic adaptive responses to limit salinity uptake, while selectively directing the metabolic advantages conferred by biopriming toward shoot establishment.

## 5. Conclusions

This study elucidates the genome-level functional potential of the salt-tolerant PGPB *Priestia aryabhattai* WJ45 and offers a comprehensive evaluation of its role in enhancing wheat seed germination under saline conditions. Notably, the genetic repertoire associated with osmotic regulation, ion homeostasis, ROS management, and secondary metabolite biosynthesis indicates that WJ45 harbors the biological mechanisms necessary to support early crop establishment in salt-stressed environments. Nevertheless, the biological validation conducted in this study was limited to the germination stage under controlled conditions, which inherently lack the complex abiotic and biotic environment present in natural soil. In such a setting, the strain does not encounter challenges such as soil impedance, fluctuating water availability, or the need for competitive colonization against indigenous microbiota. To verify the practical efficacy of WJ45 under more complex environmental conditions where both abiotic and biotic stresses are present, future investigations should involve long-term growth assessments under greenhouse and field conditions coupled with comprehensive physiological and molecular analyses of plant–microbe interactions under salt stress. These efforts are crucial for a more thorough evaluation of WJ45’s potential as a microbe-based approach to sustainable agriculture.

## Figures and Tables

**Table 1:** Plant growth-promoting traits and salt stress tolerance of WJ45.

	Plant Growth-Promoting Trait				Relative Growth Under Salt Stress (%)			
	EPS Production	Siderophore Production	Phosphate Solubilization	IAA Production	3%	5%	7%	10%
WJ45	+	+	+	+	110.5 ± 1.8 a	104.9 ± 1.8 b	104.9% ± 2.0 b	91.3 ± 1.3 c

“+” denotes a positive response in the respective plant growth-promoting trait assay. Relative growth (%) was calculated based on OD_600_ values and expressed relative to the non-salt control, which was set to 100%. Data represent the mean ± standard deviation (SD) of three independent replicates (n = 3). Statistical significance was determined using one-way ANOVA. Different letters indicate statistically significant differences (*p* < 0.05). Abbreviations: EPS, exopolysaccharide; IAA, indole-3-acetic acid

**Table 2:** Proposed mechanisms of *Priestia aryabhattai* WJ45 for enhancing wheat salt tolerance based on genomic and functional profiling.

Functional Category	Putative Mechanism	Key Genes	Functional Role in Salt Stress Tolerance
Plant Growth Promotion	Phosphate solubilization	*phoA*, *phoB*, *phoD*, *phoR*, *phoU*	Enhances phosphate availability to promote root development and plant vigor under nutrient-limited saline conditions.
	EPS production	*algA*, *pgaD*, *bcsA*	Protects seedling health by forming a physical barrier on root surfaces and improving soil moisture retention.
	Siderophore secretion	*asbA*, *entB*, *fhuBCD*, *iucA*/*C*, *sufD*	Improves iron uptake and prevents pathogen colonization to support robust plant growth and immunity.
Stress Adaptation	Ion homeostasis	*mrpA–G*, *nhaC*, *trkA*, *trkH*, *ktrD*	Mitigates ion toxicity by facilitating active Na^+^ efflux and maintaining a favorable K^+^/Na^+^ ratio.
	Osmolyte metabolism	*proABC*, *proP*, *proV*, *proWX*, *opuAB*, *opuD*	Facilitates osmotic adjustment via de novo biosynthesis and transport of compatible solutes to prevent dehydration.
	Antioxidant activity	*katA*, *katE*, *sodC*, *sodA*, *trxB*, *trxA*, *bcp*, *tpx*	Maintains redox homeostasis by scavenging ROS, thereby protecting plant cells from oxidative damage

Abbreviations: EPS, exopolysaccharidel; ROS, reactive oxygen species.

## Data Availability

The original contributions presented in this study are included in the article/Supplementary Material. Further inquiries can be directed to the corresponding author.

## Supplementary Materials

The following supporting information can be downloaded at: https://www.mdpi.com/article/10.3390/microorganisms14030605/s1. Table S1. Stability of *P. aryabhattai* WJ45 cell viability in distilled water during the priming period. Table S2. Salt stress tolerance and plant growth-promoting traits of bacterial isolates. Table S3. Detailed genomic characterization of genes associated with plant growth promotion and salt stress tolerance in *P. aryabhattai* WJ45. Table S4. Predicted biosynthetic gene clusters in the *P. aryabhattai* WJ45 genome. Table S5. Effects of *Priestia aryabhattai* WJ45 biopriming on wheat germination parameters under saline conditions. Table S6. Genomic determinants in *P. aryabhattai* WJ45 associated with plant colonization. Figure S1. Phylogenetic analysis of Priestia aryabhattai WJ45 based on 16S rRNA gene sequences. The phylogram illustrates the evolutionary relationships between WJ45 and closely related taxa. Bootstrap values, calculated from 1000 replications, are indicated at the branch points.

## Abbreviations

The following abbreviations are used in this manuscript:

PGPB	Plant growth-promoting bacteria
TSA	Tryptic soy agar
TSB	Tryptic soy broth
EPS	exopolysaccharide
IAA	Indole-3-acetic acid
WGS	Whole genome sequencing
ANI	Average nucleotide identity
DW	Distilled water
GP	Germination percentage
GI	Germination index
MGT	Mean germination time
SD	Standard deviation
ANOVA	Analysis of variance
ROS	Reactive oxygen species
BGC	Biosynthetic gene cluster
