# Supplementary material for: Whole-Genome Analysis of Priestia aryabhattai WJ45 Reveals a Genetic Repertoire Associated with Enhanced Wheat Germination and Early Seedling Growth Under Salt Stress
Source: Microorganisms. 2026 Mar 9;14(3):605. doi: 10.3390/microorganisms14030605 (PMC13028770; doi:10.3390/microorganisms14030605)
Supplement: Supplementary file 1 [file microorganisms-14-00605-s001.zip › Figure S1.pdf]

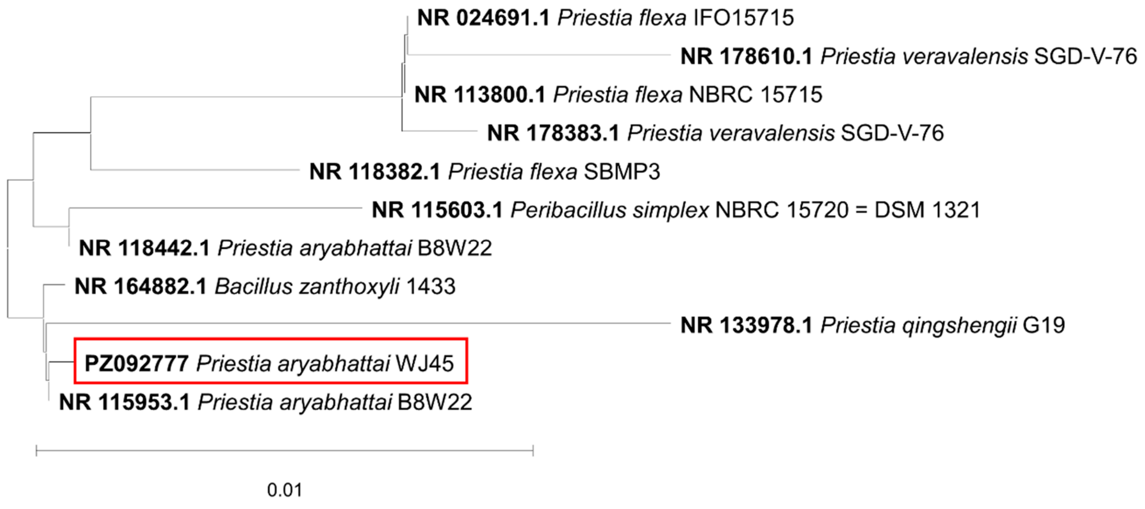

Figure S1. Phylogenetic analysis of *Priestia aryabhattai* WJ45 based on 16S rRNA gene sequences. The phylogram illustrates the evolutionary relationships between WJ45 and closely related taxa. Bootstrap values, calculated from 1000 replications, are indicated at the branch points.
